# Social contacts patterns relevant to the transmission of infectious diseases in Suzhou, China following the COVID-19 epidemic

**DOI:** 10.1186/s41043-024-00555-x

**Published:** 2024-05-09

**Authors:** Mengru Wang, Congju Wang, Guoping Gui, Feng Guo, Risheng zha, Hongpeng Sun

**Affiliations:** 1 School of Public Health, Jiangsu Key Laboratory of Preventive and Translational Medicine for Geriatric Diseases, MOE Key Laboratory of Geriatric Diseases and Immunology, Suzhou Medical College of Soochow University, Suzhou, Jiangsu 215123 P.R. China; 2 Suzhou High-tech Zone Center for Disease Control and Prevention, Suzhou, 215011 P.R. China

**Keywords:** Contact pattern, Age-structured contact matrices, Post-COVID-19 era

## Abstract

**Background:**

The COVID-19 pandemic has profoundly affected human social contact patterns, but there is limited understanding regarding the post-pandemic social contact patterns. Our objective is to quantitatively assess social contact patterns in Suzhou post-COVID-19.

**Methods:**

We employed a diary design and conducted social contact surveys from June to October 2023, utilizing paper questionnaires. A generalized linear model was utilized to analyze the relationship between individual contacts and covariates. We examined the proportions of contact type, location, duration, and frequency. Additionally, age-related mixed matrices were established.

**Results:**

The participants reported an average of 11.51 (SD 5.96) contact numbers and a total of 19.78 (SD 20.94) contact numbers per day, respectively. The number of contacts was significantly associated with age, household size, and the type of week. Compared to the 0–9 age group, those in the 10–19 age group reported a higher number of contacts (IRR = 1.12, CI: 1.01–1.24), while participants aged 20 and older reported fewer (IRR range: 0.54–0.67). Larger households (5 or more) reported more contacts (IRR = 1.09, CI: 1.01–1.18) and fewer contacts were reported on weekends (IRR = 0.95, CI: 0.90–0.99). School had the highest proportion of contact durations exceeding 4 h (49.5%) and daily frequencies (90.4%), followed by home and workplace. The contact patterns exhibited clear age-assortative mixing, with Q indices of 0.27 and 0.28.

**Conclusions:**

We assessed the characteristics of social contact patterns in Suzhou, which are essential for parameterizing models of infectious disease transmission. The high frequency and intensity of contacts among school-aged children should be given special attention, making school intervention policies a crucial component in controlling infectious disease transmission.

**Supplementary Information:**

The online version contains supplementary material available at 10.1186/s41043-024-00555-x.

## Introduction

Many infectious diseases that spread through droplets or direct contact often rely on social contacts within the population for transmission. Quantifying social contact patterns allows for a deeper understanding of the relationship between contact patterns and such infectious diseases, providing a scientific basis for prevention and control measures. The COVID-19 pandemic has had a profound impact on various societal aspects, encompassing social interactions and contact patterns. Research consistently demonstrates a significant reduction in the number of contacts during the COVID-19 pandemic compared to pre-pandemic levels [1–7]. A systematic review found that the average number of daily contacts per person during the COVID-19 pandemic ranged from 2 to 5, significantly lower compared to the pre-pandemic range of 7 to 26 contacts [8]. Juanjuan Zhang et al. conducted an estimation of contact pattern changes before, during, and after the pandemic outbreak and observed that social contacts in Shanghai, Wuhan, Shenzhen, and Changsha had recovered to 5–17% of pre-pandemic levels following the relaxation of restrictions from March to May 2020 [9]. Extensive research has been conducted on social contact patterns both before and during the COVID-19 pandemic. However, with China having implemented the “Class B Management” plan for COVID-19 infections, and all intervention measures have been fully lifted, the specific contact patterns post-COVID-19 outbreak remain unclear due to limited existing information.

Research has demonstrated significant variations in contact patterns across different income environments, with specific socio-demographic factors such as age, family structure, and employment status exerting a profound influence on the transmission dynamics of infectious diseases [10]. In Suzhou, a prominent city in the eastern region of China known for its high population density, thriving economy, and cultural diversity, distinct social and economic characteristics prevail. The dynamic population mobility, flourishing tourism industry, robust economic vitality, and extensive cultural exchanges in Suzhou make it a potential hotspot for the transmission of infectious diseases. However, despite the increasing significance of Suzhou, there is limited research available on social contact patterns specific to this region. To date, no publicly accessible data on age-specific contacts in the Suzhou area has been published.

This study aims to quantitatively analyze the social contact patterns relevant to infectious diseases in Suzhou following the COVID-19 epidemic, thereby enhancing our ability to effectively address current and future outbreaks of infectious diseases.

## Methods

### Study population

The social contact survey was conducted in the communities of Suzhou from June to July 2023 for individuals aged 20 and above, and in October 2023 for those aged 19 and below, with the written informed consent of participants. The study employed a stratified sampling method. We randomly selected eight administrative districts in Suzhou city. From each district, one community was randomly selected. Within each community, the target population was divided into eight age groups: 0–9 years, 10–19 years, 20–29 years, 30–39 years, 40–49 years, 50–59 years, 60–69 years, and 70 years and above. Participants were recruited randomly within each age group. A total of 1,608 participants were surveyed.

### Survey methodology

We employed a commonly used diary-based paper questionnaire design, which captured essential demographic information of the participants such as age, gender, occupation, education level, income, household size and other relevant factors. Additionally, for each interaction recorded in the diaries, participants were requested to provide details regarding age range, gender and contact information. A contact was defined as either physical contact such as shaking hands or hugging, or a two-way conversation with three or more words in the physical presence of another person [11, 12].

The detailed contact diary entries allowed participants to report a maximum of 20 contacts. Any contacts exceeding this limit were recorded as omissions. In addition to documenting the contacts that met the defined criteria, the student population was also required to include the number of remaining students in their class as part of the omission count. Considering the limited understanding and cognitive abilities of children, particularly those aged 0–9, the questionnaire survey was completed with the assistance of their parents. The questionnaire survey was self-reported by the participants. Trained community doctors provided guidance to ensure participants’ understanding of the questionnaire content. Participants were encouraged to record each contact as it occurred rather than waiting until the end of the day. The trained community doctors also supervised and ensured data quality to facilitate data collection.

### Statistical analysis

We examined the average daily number of contacts per respondent, considering factors such as age, sex, education level, income, household size, and day of the week. Additionally, we incorporated covariates (age, sex, education level, income, household size, and day of the week) into a generalized linear model with negative binomial regression to estimate the incidence rate ratio (IRR). To analyze the relationship between contact type, location, duration, and frequency, the contact intensity was quantified and visualized through plots depicting proportions of contact types, locations, durations, and frequencies.

To estimate the age-specific contact number per participant per day, we established age-related original and symmetrized contact matrices. The Q index was utilized to quantify the degree of departure from proportional mixing, ranging from 0 (proportional) to 1 (fully assortative) [13, 14]. We further stratified the contact matrices based on contact location, sex, contact type, and day of the week. Data analysis and visualization were conducted using SAS 9.4 and R package ggplot2, with two-sided *p* values of < 0.05 considered statistically significant.

## Results

### Demographic characteristics of participants and number of contacts

**Fig. 1:**
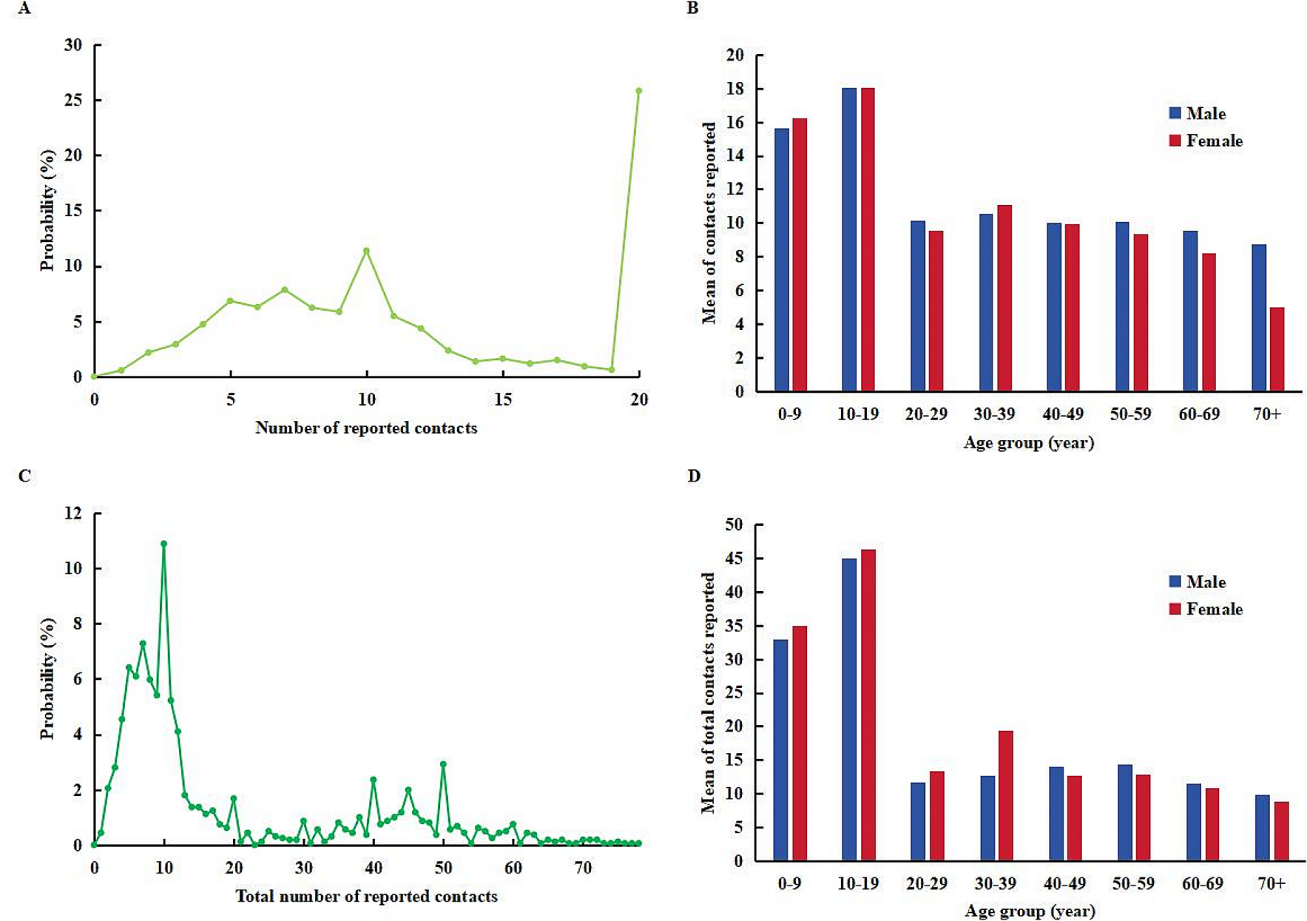
Distribution of the number of reported contacts and total contacts. (**A, B**) Correspond to the number of reported contacts; (**C, D**) Correspond to the total number of reported contacts

The final analysis included a total of 1,608 participants, 756 (47.0%) were male and 852 (53.0%) were female (Table 1). We found no differences in the age distribution and sex of participants between the recruited participants and the general population of Jiangsu Province (Supplementary Table S1). Figure 1A and B depict the distribution of contact number and total contact number. In this study, we recorded a total of 18,510 contacts, with an average of 11.51 contacts per participant per day (SD 5.96). The cumulative count of contacts reached 31,814 contacts, out of which 3,779 (24.7%) were self-reported omissions by the participants themselves, averaging 19.78 per participant per day (SD 20.94). The distribution of total contacts exhibits a significant right-skewness, with 310 participants (19.3%) reporting daily contacts exceeding 40. Furthermore, it was observed that individuals aged 10–19 had the highest number of contacts, followed by those aged 0–9. Moreover, females under the age of 39 exhibited a greater number of contacts compared to males in the same age group; however, this trend reversed among individuals above the age of 40 (Fig. 1C, D).

**Table 1:** Number of contacts and incidence rate ratios (IRRs) from the generalized linear model by demographic characteristics (*N* = 1608)

Characteristics	Number of participants *n* (%)	Mean of number of reported contacts (SD)	IRR (95% CI)^a^	Mean of total number of reported contacts (SD)	IRR (95% CI)^b^
Age, years					
0–9	178 (11.1)	15.93 (5.65)	Reference	33.93 (18.74)	Reference
10–19	229 (14.2)	18.07 (4.22)	1.12 (1.02–1.24)*	45.67 (16.95)	1.22 (1.04–1.43)*
20–29	199 (12.4)	9.85 (4.82)	0.61 (0.53–0.69)*	12.58 (11.26)	0.31 (0.25–0.38)*
30–39	204 (12.7)	10.86 (4.86)	0.67 (0.59–0.76)*	16.55 (27.09)	0.39 (0.32–0.47)*
40–49	199 (12.4)	10.02 (4.95)	0.63 (0.55–0.71)*	13.30 (12.59)	0.33 (0.27–0.40)*
50–59	195 (12.1)	9.67 (5.00)	0.61 (0.54–0.68)*	13.62 (14.45)	0.37 (0.31–0.43)*
60–69	203 (12.6)	8.83 (4.76)	0.57 (0.51–0.63)*	11.19 (17.89)	0.32 (0.27–0.38)*
70-	201 (12.5)	8.40 (5.16)	0.54 (0.49–0.60)*	9.26 (7.69)	0.28 (0.24–0.33)*
Sex					
Male	756 (47.0)	11.83 (5.94)	Reference	19.89 (17.71)	Reference
Female	852 (53.0)	11.23 (5.97)	0.97 (0.92–1.01)	19.69 (23.45)	1.06 (0.98–1.13)
Education					
Primary or below	452 (28.1)	12.38 (6.36)	Reference	22.65 (19.24)	Reference
Secondary	546 (34.0)	12.08 (6.27)	1.02 (0.95–1.10)	22.62 (22.72)	1.14 (1.02–1.28)*
Post-secondary	610 (37.9)	10.36 (5.15)	1.05 (0.95–1.16)	15.12 (19.64)	1.29 (1.11–1.50)*
Income, CNY					
0–5,999	1022 (63.6)	12.37 (6.36)	Reference	23.18 (23.35)	Reference
6,000–9,999	361 (22.5)	10.21 (5.04)	1.02 (0.95–1.10)	13.48 (13.13)	1.02 (0.92–1.13)
10,000–14,999	160 (10.0)	10.10 (4.65)	1.01 (0.92–1.10)	14.94 (16.07)	1.08 (0.95–1.24)
>=15,000	65 (4.0)	8.72 (4.20)	0.86 (0.75–0.98)*	13.35 (13.87)	1.00 (0.83–1.22)
Household size					
1–2	203 (12.6)	8.98 (5.73)	Reference	11.41 (11.70)	Reference
3–4	802 (49.9)	11.51 (6.04)	1.06 (0.98–1.14)	19.89 (18.67)	1.15 (1.03–1.30)*
5+	603 (37.5)	12.37 (5.70)	1.09 (1.01–1.18)*	22.47 (25.10)	1.20 (1.06–1.35)*
Day of the week					
Weekdays	1165 (72.5)	12.09 (6.20)	Reference	22.42 (22.83)	Reference
Weekends	443 (27.5)	9.98 (4.99)	0.95 (0.90–0.99)*	12.85 (12.47)	0.78 (0.72–0.84)*

^a^Dispersion parameter alpha = 1.12 (95% CI 1.11–1.14)

^b^Dispersion parameter alpha = 1.52 (95% CI 1.48–1.58)

IRR: incidence rate ratio

Adjusted for age, sex, education, income, household size and day of the week

Our analysis revealed significant associations between the number of contacts and age, household size, and day of the week (Table 1). Compared to individuals aged 0–9, those in the 10–19 age group exhibited a higher number of contacts (IRR = 1.12, CI: 1.01–1.24), while participants aged 20 and above had fewer contacts with IRRs ranging from 0.54 to 0.67. Moreover, participants residing in households with a size of five or more reported a greater number of contacts (IRR = 1.09, CI: 1.01–1.18), whereas fewer contacts were reported on weekends by all participants (IRR = 0.95, CI: 0.90-0 0.99). The total number of contacts, accounting for omissions, also exhibits significant associations with age, household size, and the day of the week.

### Type, location, duration and frequency of contacts

**Fig. 2:**
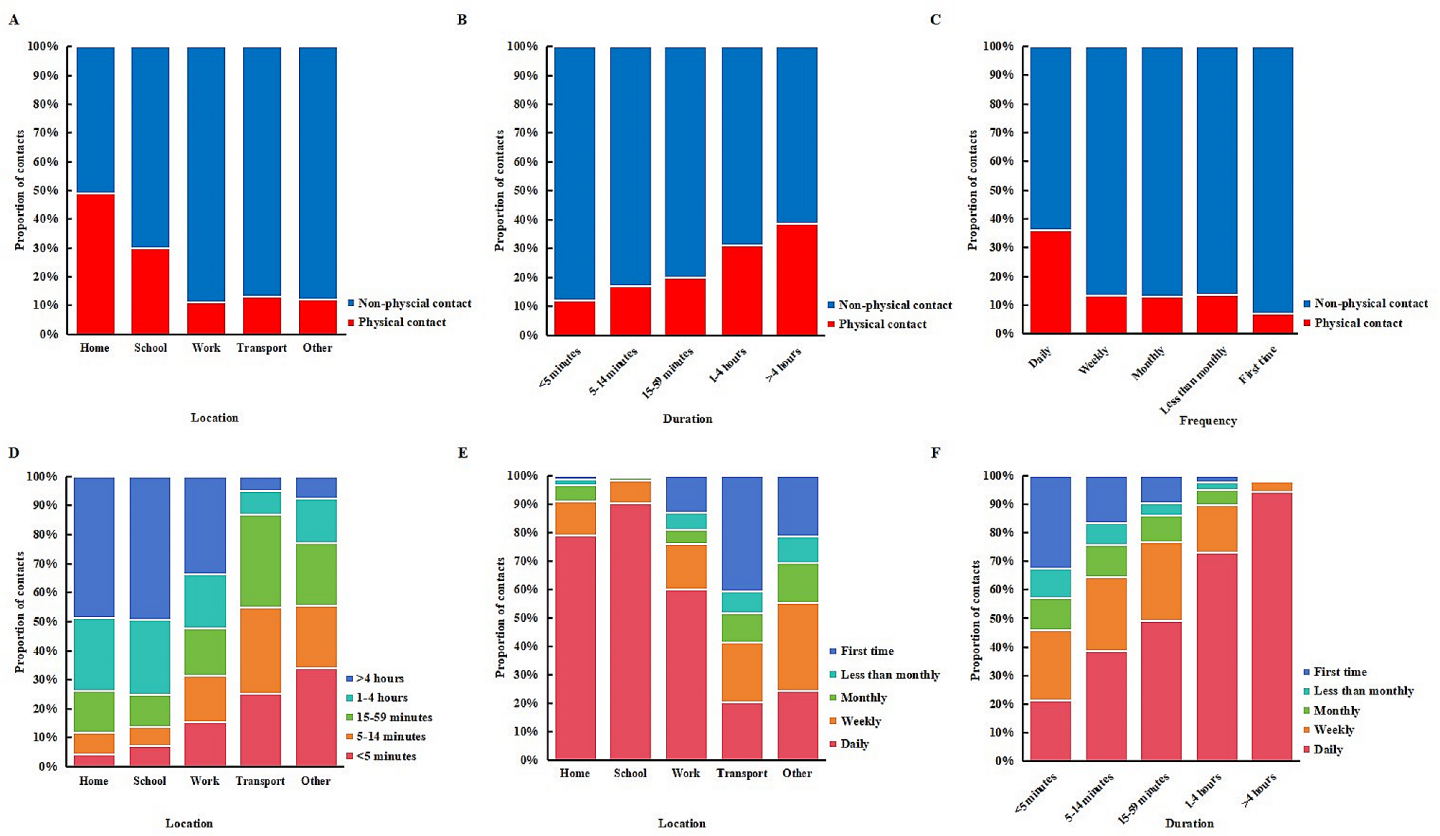
The relationship between contact type, location, duration and frequency. The proportion of contacts that were physical or non-physical by (**A**) location, (**B**) duration and (**C**) frequency of contacts. The proportion between (**D**) location and frequency, (**E**) location and duration, and (**F**) duration and frequency of contacts

Figure 2 illustrates that physical contact is more prevalent at home (48.9%), followed by school (29.9%). Moreover, as the duration and frequency of contact increase, so does the likelihood of physical interaction. First-time contacts and contacts lasting less than 5 min involved fewer instances of physical contact. Approximately half of all school-based contacts exceed four hours in length (49.5%), followed by contacts at home (48.7%) and in the work (33.6%). Daily occurrences are most common among school-related contacts (90.4%), followed by those at home (78.9%) and work (60.1%). The frequency of contacts increased with longer durations of contact. The vast majority (94.5%) of contacts lasting longer than four hours occur on a daily basis.

### Age-related contact matrix

**Fig. 3:**
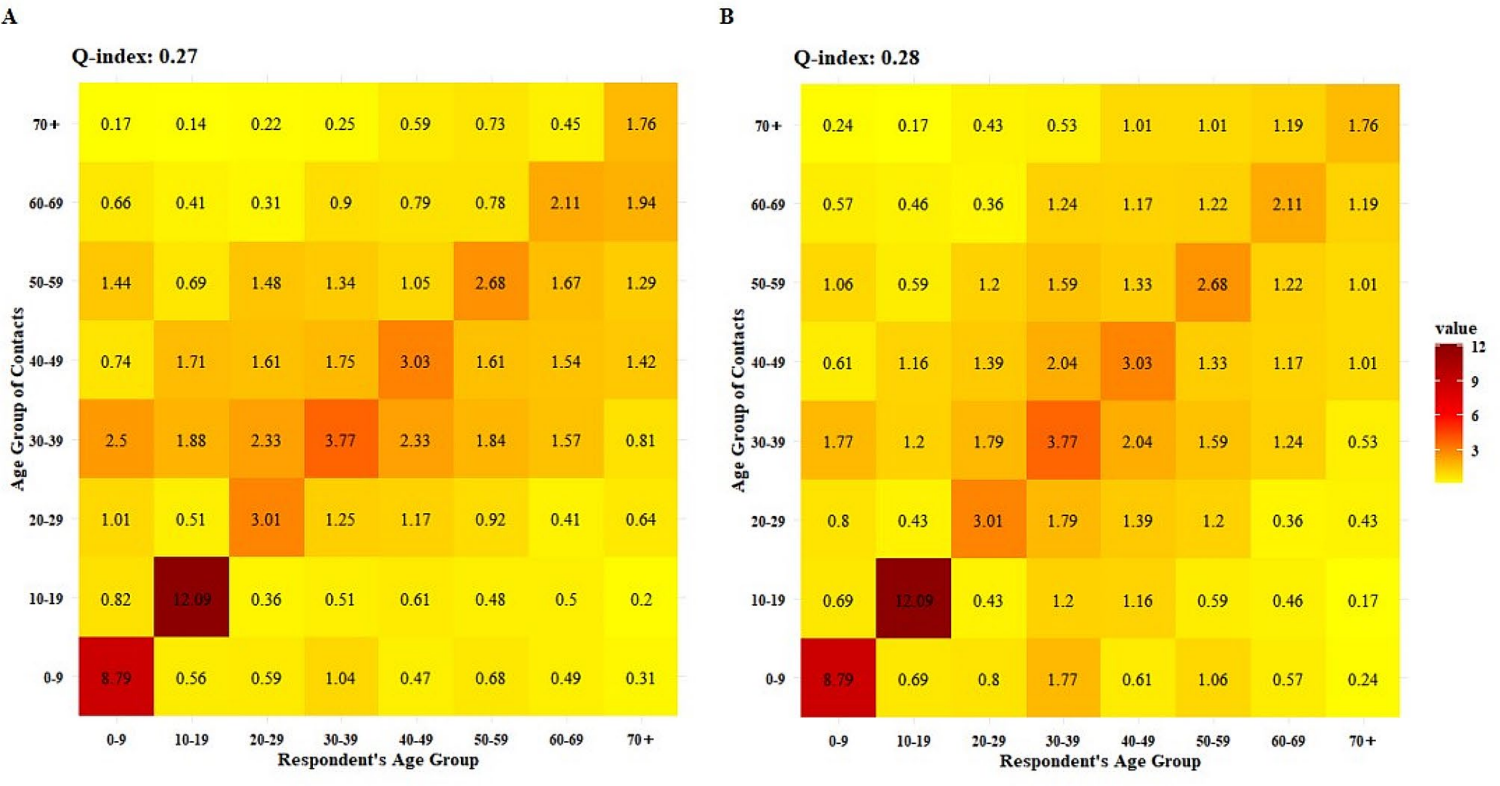
Contact matrix of reported contacts consisting of average number of contacts per day per participant. (**A**) Original contact matrice; (**B**) Symmetrized contact matrice. The symmetrized contact matrices are calculated using ${c}_{ij}=\frac{{c}_{ij}+{c}_{ji}}{2}$

The age-specific assortative mixing pattern is clearly demonstrated in Fig. 3, with a distinct central diagonal line. The Q indices for the original and symmetrized contact matrices are 0.27 and 0.28 respectively, indicating a strong level of assortativity. Among all age groups, individuals aged 10–19 exhibit the most pronounced pattern, while those aged 70 and above show the least evident pattern. Participants and contacts displayed two parallel secondary diagonals starting from the age group of 30–39, indicating intergenerational contact mixing, primarily within familial relationships (Supplementary Figure S1). Within the age range of 20–59, there was a relatively diverse array of contacts observed, particularly in occupational settings (Supplementary Figure S3). After stratification by sex, contact type, and day type, no significant changes were observed except for the absence of contact features among working-age adults in the physical contact matrix (Supplementary Figure S5-10).

## Discussion

We conducted a social contact survey among 1,608 individuals in Suzhou City following the COVID-19 pandemic. Our findings revealed that the patterns of social contact exhibited overall consistency with those observed in Europe and other regions worldwide prior to the pandemic. Following the COVID-19 pandemic, we estimated that residents in Suzhou had an average of 19.78 daily contacts, similar to the numbers reported in Shanghai (18.7) [15], Guangdong Province (16.7) [16], and Southern China (18.6) [17] prior to the outbreak. Suzhou, Shanghai, Guangdong Province, and Southern China share similar geographical environments, economies, cultures and pandemic policies. They are all located in southern China along the eastern coast, with comparable climatic conditions and well-developed transportation networks. These regions are culturally open with rich historical heritage, representing significant aspects of Chinese culture. Moreover, they serve as vital engines of the Chinese economy. Hence, it can be tentatively inferred that the level of social interaction in China has roughly returned to pre-pandemic levels.

We observed variations in the number of contacts based on age, household size, and day of the week, which aligns with previous research findings [1, 18–20]. However, unlike some countries where individuals of working-age tend to have the highest number of contacts while those aged 0–9 have relatively fewer contacts, our study revealed that children aged 0–19 had the highest number of contacts. This discrepancy may be attributed to early enrollment in childcare facilities and comparatively larger class sizes among students in Suzhou, China.

The spread of infectious diseases within a population is influenced by various factors, but the primary factor is the susceptibility of individuals within the population. The intensity of contact, duration of contact, and frequency of contact with infected individuals directly determine the risk of susceptibility for susceptible individuals. In theory, the greater the intensity of contact, the longer the duration, and the more frequent the contact, the higher the risk of infection [21]. Home and school have been convincingly demonstrated as high-intensity contact environments [11, 12, 22, 23]. Our study further reveals that the proportion of contact duration exceeding four hours is highest at school rather than at home. Moreover, daily contacts are predominantly made at school followed by home. These findings suggest that in Suzhou City, children aged 0–19 years old have longer and more frequent contacts at school compared to home. By recognizing the heightened and prolonged exposure to potential pathogens in school environments, public health authorities can implement targeted interventions to reduce the spread of infectious diseases. Strategies such as improving ventilation, implementing regular disinfection protocols, and promoting hygiene practices within schools can play a crucial role in lowering transmission rates. Additionally, educational campaigns aimed at students, teachers, and parents can enhance awareness of preventive measures and encourage adherence, ultimately ensuring the overall health of the community.

The contact matrix patterns identified in existing research primarily exhibit three characteristics. Firstly, there is a propensity for individuals to gravitate towards contacts with peers, which is most pronounced among school-age children and least discernible among the elderly [24–27]. Furthermore, there exists intergenerational mixed contact encompassing interactions between offspring and their parents or (grand) parents within the familial context [23, 28, 29]. Thirdly, the working-age population typically maintains extensive interpersonal connections within their professional environments [30, 31]. The observed contact patterns closely align with global findings. The key distinction between the latter two characteristics lies in the initial age. In comparison Japan [27], Vietnam [28] and Cambodia [30], we observed a relatively higher initial age, with a disparity of 5–10 years. However, our age distribution exhibits similarities to that found in European countries [11].

Our study also has certain limitations. Firstly, we employed paper-based questionnaires, which significantly improved the response rate and facilitated better quality control. However, due to a limitation in the survey questionnaire that restricted reporting individual contacts to a maximum of 20, there might have been potential underreporting despite incorporating omission count as an additional measure, thereby introducing slight biases. Secondly, relying solely on self-reporting for the contact survey introduces the possibility of recall bias and self-report bias. While community healthcare workers encouraged participants to prospectively record all their contacts whenever possible, we cannot guarantee strict adherence to this requirement by every participant. Self-reporting may lead participants to forget recording encounters with less familiar individuals who have shorter durations and lower frequencies, thereby underestimating the number of contacts. In the future, this can be avoided through prospective designs and advanced data collection methods employing sophisticated equipment. Thirdly, considering children’s limited understanding and cognitive abilities, parental assistance was sought while completing the questionnaire, particularly for those in primary school or younger, however, this approach may introduce reporting biases in our survey results. Fourthly, this study did not account for potential confounding variables such as such as cultural practices, community norms, or recent events beyond the COVID-19 pandemic that could influence social contact patterns. More research is necessary to evaluate how these social factors affect the number of social contacts.

## Conclusions

In conclusion, we conducted an assessment of the characteristics of social contacts and mixing patterns relevant to the transmission of infectious diseases in Suzhou, following the COVID-19 pandemic. We estimated that residents in Suzhou have an average of 19.78 daily contacts per person, indicating a relatively high level of contact. The obtained age mixing patterns provide valuable insights for parameterizing dynamic disease transmission in this region. Notably, particular attention should be given to the heightened frequency and intensity of interactions among school-age children. Therefore, implementing intervention measures within educational settings during infectious disease outbreaks will be effective.

### Electronic supplementary material

Below is the link to the electronic supplementary material.

Supplementary Material 1

Supplementary Material 2

Supplementary Material 3

## Data Availability

All data generated or analysed during this study are included in this published article.
