# Supplementary material for: Social contacts patterns relevant to the transmission of infectious diseases in Suzhou, China following the COVID-19 epidemic
Source: J Health Popul Nutr. 2024 May 9;43:58. doi: 10.1186/s41043-024-00555-x (PMC11080078; doi:10.1186/s41043-024-00555-x)
Supplement: Supplementary file 1 — Supplementary Material 1 [file 41043_2024_555_MOESM1_ESM.docx]

Table S1. Comparison between all of the participants and the population of Jiangsu Province.

| **Characteristics** | **Population of Jiangsu Province (%)**  **N=340,670** | **Participants (%)**  **N=1,608** | **χ^2^** | **P** |
| --- | --- | --- | --- | --- |
| **Sex** |  |  |  |  |
| **Male** | 170282 (50.0) | 756 (47.0) | 5.645 | 0.0175 |
| **Female** | 170388 (50.0) | 852 (53.0) |  |  |
| **Age** |  |  |  |  |
| **0-9** | 31823 (9.3) | 178 (11.1) | 106.142 | <0.001 |
| **10-19** | 29331 (8.6) | 229 (14.2) |  |  |
| **20-29** | 38572 (11.3) | 199 (12.4) |  |  |
| **30-39** | 48551 (14.3) | 204 (12.7) |  |  |
| **40-49** | 51844 (15.2) | 199 (12.4) |  |  |
| **50-59** | 58521 (17.2) | 195 (12.1) |  |  |
| **60-69** | 45623 (13.4) | 203 (12.6) |  |  |
| **70＋** | 36405 (10.7) | 201 (12.5) |  |  |

The demographic data for Jiangsu Province represents a sample from the 2021 population census, with a sampling ratio of 0.44%.

Table S2. Contact matrix reported contacts consisting of the mean number of contacts per day per participant

|  | | **Age of contact** | | | | | | | |
| --- | --- | --- | --- | --- | --- | --- | --- | --- | --- |
|  |  | **0-9** | **10-19** | **20-29** | **30-39** | **40-49** | **50-59** | **60-69** | **>=70** |
| **Age of participant** | **0-9** | 8.79 | 0.82 | 1.01 | 2.5 | 0.74 | 1.44 | 0.66 | 0.17 |
|  | **10-19** | 0.56 | 12.09 | 0.51 | 1.88 | 1.71 | 0.69 | 0.41 | 0.14 |
|  | **20-29** | 0.59 | 0.36 | 3.01 | 2.33 | 1.61 | 1.48 | 0.31 | 0.22 |
|  | **30-39** | 1.04 | 0.51 | 1.25 | 3.77 | 1.75 | 1.34 | 0.90 | 0.25 |
|  | **40-49** | 0.47 | 0.61 | 1.17 | 2.33 | 3.03 | 1.05 | 0.79 | 0.59 |
|  | **50-59** | 0.68 | 0.48 | 0.92 | 1.84 | 1.61 | 2.68 | 0.78 | 0.73 |
|  | **60-69** | 0.49 | 0.50 | 0.41 | 1.57 | 1.54 | 1.67 | 2.11 | 0.45 |
|  | **>=70** | 0.31 | 0.20 | 0.64 | 0.81 | 1.42 | 1.29 | 1.94 | 1.76 |

Table S3. Symmetrized contact matrix contacts consisting of the mean number of contacts per day per participant

|  | | **Age of contact** | | | | | | | |
| --- | --- | --- | --- | --- | --- | --- | --- | --- | --- |
|  |  | **0-9** | **10-19** | **20-29** | **30-39** | **40-49** | **50-59** | **60-69** | **>=70** |
| **Age of participant** | **0-9** | 8.79 | 0.69 | 0.80 | 1.77 | 0.61 | 1.06 | 0.57 | 0.24 |
|  | **10-19** | 0.69 | 12.09 | 0.43 | 1.20 | 1.16 | 0.59 | 0.46 | 0.17 |
|  | **20-29** | 0.80 | 0.43 | 3.01 | 1.79 | 1.39 | 1.20 | 0.36 | 0.43 |
|  | **30-39** | 1.77 | 1.20 | 1.79 | 3.77 | 2.04 | 1.59 | 1.24 | 0.53 |
|  | **40-49** | 0.61 | 1.16 | 1.39 | 2.04 | 3.03 | 1.33 | 1.17 | 1.01 |
|  | **50-59** | 1.06 | 0.59 | 1.20 | 1.59 | 1.33 | 2.68 | 1.22 | 1.01 |
|  | **60-69** | 0.57 | 0.46 | 0.36 | 1.24 | 1.17 | 1.22 | 2.11 | 1.19 |
|  | **>=70** | 0.24 | 0.17 | 0.43 | 0.53 | 1.01 | 1.01 | 1.19 | 1.76 |

Table S4. Contact matrix of reported contacts consisting of the mean number of contacts per day per participant in home.

|  | | **Age of contact** | | | | | | | |
| --- | --- | --- | --- | --- | --- | --- | --- | --- | --- |
|  |  | **0-9** | **10-19** | **20-29** | **30-39** | **40-49** | **50-59** | **60-69** | **>=70** |
| **Age of participant** | **0-9** | 0.53 | 0.19 | 0.31 | 1.42 | 0.33 | 0.99 | 0.49 | 0.16 |
|  | **10-19** | 0.23 | 0.24 | 0.10 | 0.78 | 1.11 | 0.19 | 0.42 | 0.15 |
|  | **20-29** | 0.24 | 0.13 | 0.56 | 0.25 | 0.51 | 1.03 | 0.14 | 0.22 |
|  | **30-39** | 0.76 | 0.34 | 0.16 | 0.92 | 0.24 | 0.70 | 0.58 | 0.16 |
|  | **40-49** | 0.31 | 0.55 | 0.38 | 0.26 | 0.85 | 0.20 | 0.43 | 0.42 |
|  | **50-59** | 0.43 | 0.10 | 0.53 | 0.69 | 0.26 | 0.86 | 0.14 | 0.49 |
|  | **60-69** | 0.44 | 0.44 | 0.16 | 0.86 | 0.56 | 0.32 | 0.88 | 0.22 |
|  | **>=70** | 0.25 | 0.18 | 0.39 | 0.48 | 0.78 | 0.76 | 0.49 | 0.89 |

Table S5. Contact matrix of reported contacts consisting of the mean number of contacts per day per participant in school.

|  | | **Age of contact** | | | | | | | |
| --- | --- | --- | --- | --- | --- | --- | --- | --- | --- |
|  |  | **0-9** | **10-19** | **20-29** | **30-39** | **40-49** | **50-59** | **60-69** | **>=70** |
| **Age of participant** | **0-9** | 10.28 | 0.68 | 0.78 | 1.01 | 0.34 | 0.09 | 0.02 | 0.01 |
|  | **10-19** | 0.34 | 12.68 | 0.29 | 1.09 | 0.68 | 0.25 | 0.01 | 0.00 |
|  | **20-29** | 0.07 | 0.04 | 0.37 | 0.33 | 0.22 | 0.07 | 0.04 | 0.00 |
|  | **30-39** | 0.08 | 0.00 | 0.08 | 0.58 | 0.17 | 0.33 | 0.00 | 0.00 |
|  | **40-49** | 0.00 | 0.00 | 0.14 | 0.29 | 0.57 | 0.14 | 0.14 | 0.00 |
|  | **50-59** | 0.00 | 0.00 | 0.20 | 0.00 | 0.40 | 0.40 | 0.00 | 0.00 |
|  | **60-69** | 0.00 | 0.00 | 0.00 | 0.00 | 0.00 | 0.00 | 0.00 | 0.00 |
|  | **>=70** | 0.00 | 0.00 | 0.00 | 0.00 | 1.00 | 0.00 | 0.00 | 0.00 |

Table S6. Contact matrix of reported contacts consisting of the mean number of contacts per day per participant in workplace.

|  | | **Age of contact** | | | | | | | |
| --- | --- | --- | --- | --- | --- | --- | --- | --- | --- |
|  |  | **0-9** | **10-19** | **20-29** | **30-39** | **40-49** | **50-59** | **60-69** | **>=70** |
| **Age of participant** | **0-9** | 1.00 | 0.00 | 0.00 | 0.00 | 0.00 | 0.00 | 0.00 | 0.00 |
|  | **10-19** | 0.00 | 0.00 | 0.80 | 1.00 | 1.00 | 0.20 | 0.00 | 0.00 |
|  | **20-29** | 0.49 | 0.23 | 1.73 | 2.05 | 1.12 | 0.60 | 0.24 | 0.04 |
|  | **30-39** | 0.22 | 0.11 | 1.01 | 2.83 | 1.52 | 0.47 | 0.27 | 0.06 |
|  | **40-49** | 0.13 | 0.03 | 0.73 | 2.01 | 2.06 | 0.66 | 0.28 | 0.14 |
|  | **50-59** | 0.26 | 0.54 | 0.35 | 1.03 | 1.34 | 1.35 | 0.38 | 0.10 |
|  | **60-69** | 0.08 | 0.05 | 0.44 | 1.00 | 1.17 | 1.33 | 0.62 | 0.06 |
|  | **>=70** | 0.13 | 0.00 | 0.43 | 0.21 | 0.98 | 0.23 | 0.51 | 0.32 |

Table S7. Contact matrix of reported contacts consisting of the mean number of contacts per day per participant in transport.

|  | | **Age of contact** | | | | | | | |
| --- | --- | --- | --- | --- | --- | --- | --- | --- | --- |
|  |  | **0-9** | **10-19** | **20-29** | **30-39** | **40-49** | **50-59** | **60-69** | **>=70** |
| **Age of participant** | **0-9** | 0.33 | 0.07 | 0.20 | 0.33 | 0.20 | 0.27 | 0.13 | 0.00 |
|  | **10-19** | 0.15 | 0.59 | 0.70 | 0.70 | 0.48 | 0.33 | 0.07 | 0.00 |
|  | **20-29** | 0.07 | 0.13 | 0.53 | 0.60 | 0.40 | 0.24 | 0.07 | 0.00 |
|  | **30-39** | 0.16 | 0.11 | 0.22 | 0.57 | 0.51 | 0.30 | 0.16 | 0.05 |
|  | **40-49** | 0.00 | 0.06 | 0.19 | 0.61 | 0.58 | 0.32 | 0.13 | 0.00 |
|  | **50-59** | 0.06 | 0.04 | 0.21 | 0.32 | 0.60 | 0.57 | 0.36 | 0.17 |
|  | **60-69** | 0.00 | 0.06 | 0.09 | 0.24 | 0.55 | 0.45 | 0.70 | 0.03 |
|  | **>=70** | 0.11 | 0.05 | 0.32 | 0.11 | 0.84 | 0.43 | 0.59 | 0.14 |

Table S8. Contact matrix of reported contacts consisting of the mean number of contacts per day per participant among males.

|  | | **Age of contact** | | | | | | | |
| --- | --- | --- | --- | --- | --- | --- | --- | --- | --- |
|  |  | **0-9** | **10-19** | **20-29** | **30-39** | **40-49** | **50-59** | **60-69** | **>=70** |
| **Age of participant** | **0-9** | 8.80 | 0.59 | 1.02 | 2.58 | 0.68 | 1.34 | 0.84 | 0.13 |
|  | **10-19** | 0.67 | 11.88 | 0.51 | 1.98 | 1.90 | 0.54 | 0.45 | 0.14 |
|  | **20-29** | 0.30 | 0.29 | 3.71 | 2.66 | 1.74 | 1.24 | 0.17 | 0.18 |
|  | **30-39** | 0.87 | 0.42 | 1.23 | 4.02 | 1.63 | 1.43 | 0.72 | 0.24 |
|  | **40-49** | 0.51 | 0.80 | 1.16 | 2.38 | 2.86 | 0.92 | 0.89 | 0.57 |
|  | **50-59** | 0.68 | 0.32 | 1.09 | 1.79 | 1.83 | 2.82 | 0.61 | 0.90 |
|  | **60-69** | 0.54 | 0.40 | 0.58 | 1.73 | 1.54 | 1.85 | 2.19 | 0.52 |
|  | **>=70** | 0.33 | 0.18 | 0.70 | 0.83 | 1.60 | 1.35 | 2.09 | 1.73 |

Table S9. Contact matrix of reported contacts consisting of the mean number of contacts per day per participant among females.

|  | | **Age of contact** | | | | | | | |
| --- | --- | --- | --- | --- | --- | --- | --- | --- | --- |
|  |  | **0-9** | **10-19** | **20-29** | **30-39** | **40-49** | **50-59** | **60-69** | **>=70** |
| **Age of participant** | **0-9** | 19.32 | 1.80 | 2.22 | 5.49 | 1.63 | 3.17 | 1.44 | 0.38 |
|  | **10-19** | 1.10 | 24.07 | 1.03 | 3.73 | 3.44 | 1.37 | 0.82 | 0.27 |
|  | **20-29** | 1.11 | 0.69 | 5.70 | 4.41 | 3.05 | 2.80 | 0.59 | 0.42 |
|  | **30-39** | 1.81 | 0.89 | 2.17 | 6.52 | 3.02 | 2.32 | 1.56 | 0.44 |
|  | **40-49** | 0.87 | 1.13 | 2.16 | 4.29 | 5.58 | 1.94 | 1.46 | 1.08 |
|  | **50-59** | 1.26 | 0.90 | 1.70 | 3.41 | 2.99 | 4.98 | 1.48 | 1.35 |
|  | **60-69** | 0.90 | 0.93 | 0.76 | 2.90 | 2.84 | 3.08 | 3.90 | 0.84 |
|  | **>=70** | 0.58 | 0.38 | 1.18 | 1.50 | 2.62 | 2.39 | 3.57 | 3.24 |

Table S10. Physical contact matrix of reported contacts consisting of the mean number of contacts per day per participant.

|  | | **Age of contact** | | | | | | | |
| --- | --- | --- | --- | --- | --- | --- | --- | --- | --- |
|  |  | **0-9** | **10-19** | **20-29** | **30-39** | **40-49** | **50-59** | **60-69** | **>=70** |
| **Age of participant** | **0-9** | 2.86 | 0.36 | 0.52 | 1.62 | 0.34 | 0.87 | 0.40 | 0.11 |
|  | **10-19** | 0.35 | 3.86 | 0.11 | 0.73 | 0.69 | 0.17 | 0.17 | 0.03 |
|  | **20-29** | 0.32 | 0.06 | 0.69 | 0.32 | 0.25 | 0.26 | 0.04 | 0.03 |
|  | **30-39** | 0.74 | 0.22 | 0.19 | 0.72 | 0.21 | 0.15 | 0.11 | 0.04 |
|  | **40-49** | 0.29 | 0.34 | 0.22 | 0.32 | 0.75 | 0.17 | 0.13 | 0.13 |
|  | **50-59** | 0.39 | 0.12 | 0.17 | 0.26 | 0.21 | 0.61 | 0.06 | 0.14 |
|  | **60-69** | 0.28 | 0.17 | 0.09 | 0.26 | 0.19 | 0.19 | 0.63 | 0.18 |
|  | **>=70** | 0.16 | 0.09 | 0.11 | 0.15 | 0.19 | 0.22 | 0.30 | 0.47 |

Table S11. Non-physical contact matrix of reported contacts consisting of the mean number of contacts per day per participant.

|  | | **Age of contact** | | | | | | | |
| --- | --- | --- | --- | --- | --- | --- | --- | --- | --- |
|  |  | **0-9** | **10-19** | **20-29** | **30-39** | **40-49** | **50-59** | **60-69** | **>=70** |
| **Age of participant** | **0-9** | 5.93 | 0.46 | 0.49 | 0.88 | 0.40 | 0.57 | 0.25 | 0.06 |
|  | **10-19** | 0.21 | 8.23 | 0.40 | 1.15 | 1.03 | 0.53 | 0.24 | 0.11 |
|  | **20-29** | 0.27 | 0.30 | 2.32 | 2.01 | 1.36 | 1.22 | 0.28 | 0.20 |
|  | **30-39** | 0.30 | 0.29 | 1.06 | 3.05 | 1.53 | 1.19 | 0.79 | 0.22 |
|  | **40-49** | 0.18 | 0.28 | 0.95 | 2.01 | 2.28 | 0.88 | 0.67 | 0.46 |
|  | **50-59** | 0.28 | 0.36 | 0.74 | 1.58 | 1.41 | 2.07 | 0.72 | 0.58 |
|  | **60-69** | 0.21 | 0.33 | 0.32 | 1.32 | 1.34 | 1.48 | 1.48 | 0.28 |
|  | **>=70** | 0.15 | 0.11 | 0.53 | 0.66 | 1.23 | 1.07 | 1.63 | 1.28 |

Table S12. Contact matrix of reported contacts consisting of the mean number of contacts per day per participant in weekday.

|  | | **Age of contact** | | | | | | | |
| --- | --- | --- | --- | --- | --- | --- | --- | --- | --- |
|  |  | **0-9** | **10-19** | **20-29** | **30-39** | **40-49** | **50-59** | **60-69** | **>=70** |
| **Age of participant** | **0-9** | 9.30 | 0.73 | 1.04 | 2.42 | 0.75 | 1.30 | 0.56 | 0.16 |
|  | **10-19** | 0.56 | 12.62 | 0.48 | 1.94 | 1.80 | 0.48 | 0.41 | 0.11 |
|  | **20-29** | 0.48 | 0.31 | 3.11 | 2.25 | 1.69 | 1.46 | 0.35 | 0.24 |
|  | **30-39** | 1.00 | 0.52 | 1.06 | 3.87 | 1.81 | 1.40 | 0.90 | 0.30 |
|  | **40-49** | 0.55 | 0.56 | 1.33 | 2.41 | 3.14 | 1.01 | 0.92 | 0.61 |
|  | **50-59** | 0.78 | 0.58 | 0.88 | 1.76 | 1.76 | 2.78 | 0.78 | 0.68 |
|  | **60-69** | 0.51 | 0.47 | 0.48 | 1.45 | 1.61 | 1.62 | 2.06 | 0.48 |
|  | **>=70** | 0.28 | 0.16 | 0.66 | 0.80 | 1.47 | 1.30 | 2.01 | 1.88 |

Table S13. Contact matrix of reported contacts consisting of the mean number of contacts per day per participant in weekend.

|  | | **Age of contact** | | | | | | | |
| --- | --- | --- | --- | --- | --- | --- | --- | --- | --- |
|  |  | **0-9** | **10-19** | **20-29** | **30-39** | **40-49** | **50-59** | **60-69** | **>=70** |
| **Age of participant** | **0-9** | 4.80 | 1.55 | 0.75 | 3.10 | 0.65 | 2.55 | 1.40 | 0.30 |
|  | **10-19** | 0.59 | 9.79 | 0.86 | 1.55 | 1.45 | 2.21 | 0.48 | 0.60 |
|  | **20-29** | 0.80 | 0.46 | 2.83 | 2.46 | 1.45 | 1.51 | 0.23 | 0.19 |
|  | **30-39** | 1.14 | 0.49 | 1.70 | 3.54 | 1.60 | 1.21 | 0.90 | 0.14 |
|  | **40-49** | 0.29 | 0.73 | 0.82 | 2.15 | 2.79 | 1.13 | 0.52 | 0.53 |
|  | **50-59** | 0.45 | 0.27 | 1.00 | 2.02 | 1.27 | 2.47 | 0.78 | 0.83 |
|  | **60-69** | 0.45 | 0.55 | 0.30 | 1.78 | 1.41 | 1.75 | 2.20 | 0.41 |
|  | **>=70** | 0.38 | 0.30 | 0.61 | 0.84 | 1.33 | 1.28 | 1.78 | 1.50 |
