# Supplementary material for: Social contacts patterns relevant to the transmission of infectious diseases in Suzhou, China following the COVID-19 epidemic
Source: J Health Popul Nutr. 2024 May 9;43:58. doi: 10.1186/s41043-024-00555-x (PMC11080078; doi:10.1186/s41043-024-00555-x)

Supplementary figures

Figure S1. Contact matrix of reported contacts consisting of the average number of contacts per day per participant in home.


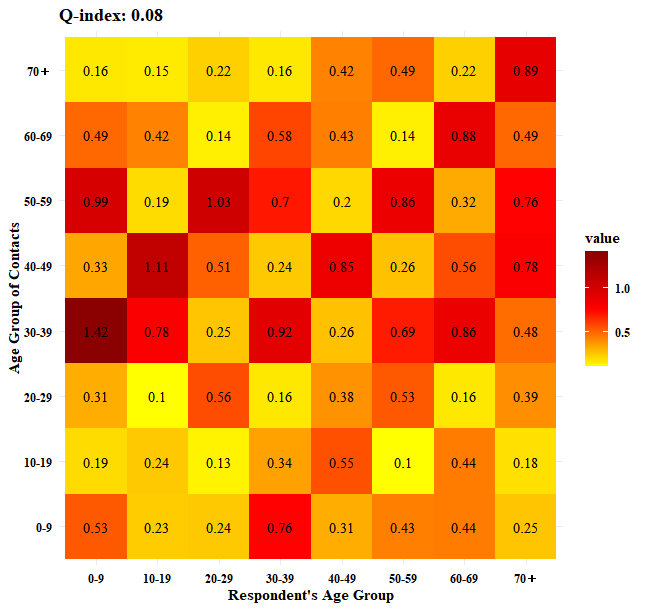


Figure S2. Contact matrix of reported contacts consisting of the average number of contacts per day per participant in school.


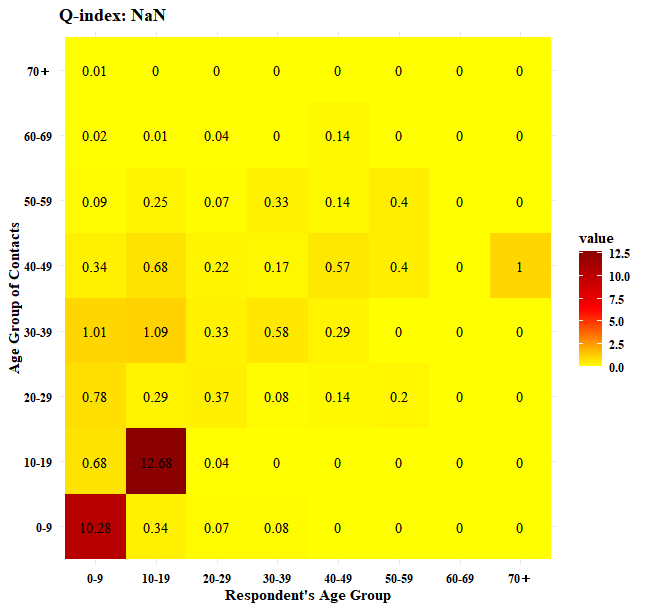


Figure S3. Contact matrix of reported contacts consisting of the average number of contacts per day per participant in workplace.


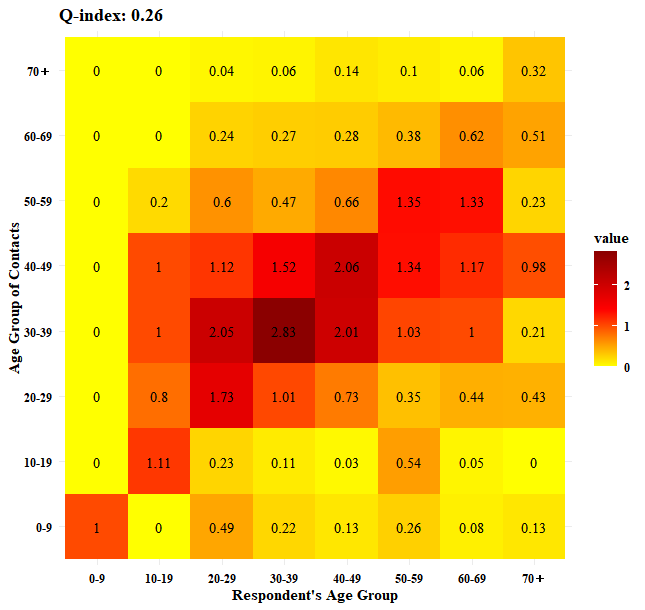


Figure S4. Contact matrix of reported contacts consisting of the average number of contacts per day per participant in transport.


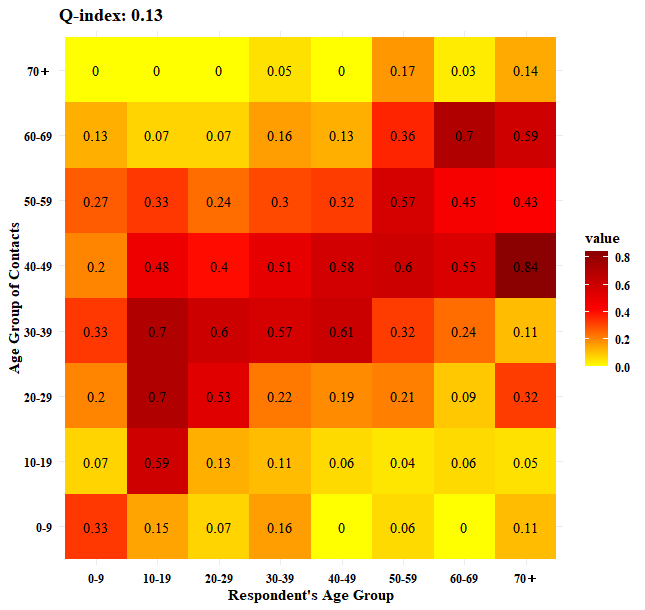


Figure S5. Contact matrix of reported contacts consisting of the average number of contacts per day per participant among males.


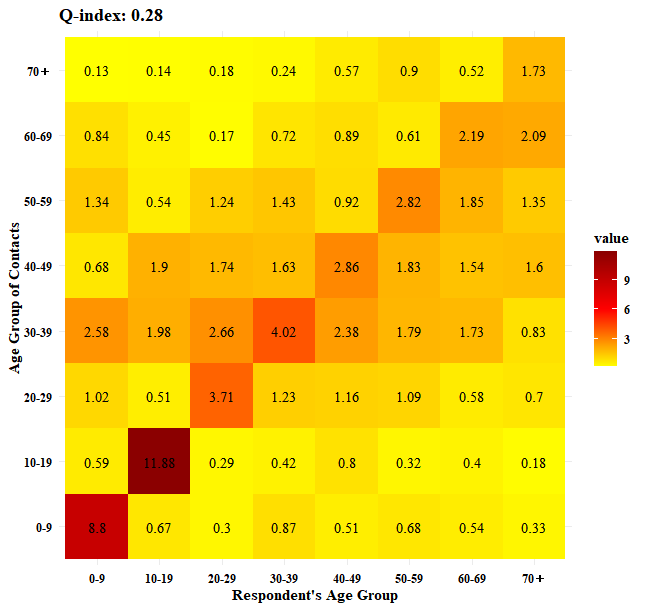


Figure S6. Contact matrix of reported contacts consisting of the average number of contacts per day per participant among females.


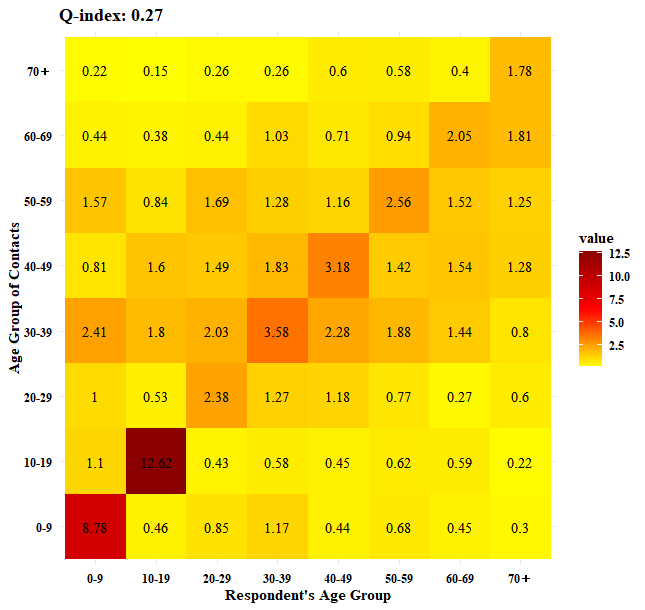


Figure S7. Physical contact matrix of reported contacts consisting of the average number of contacts per day per participant.


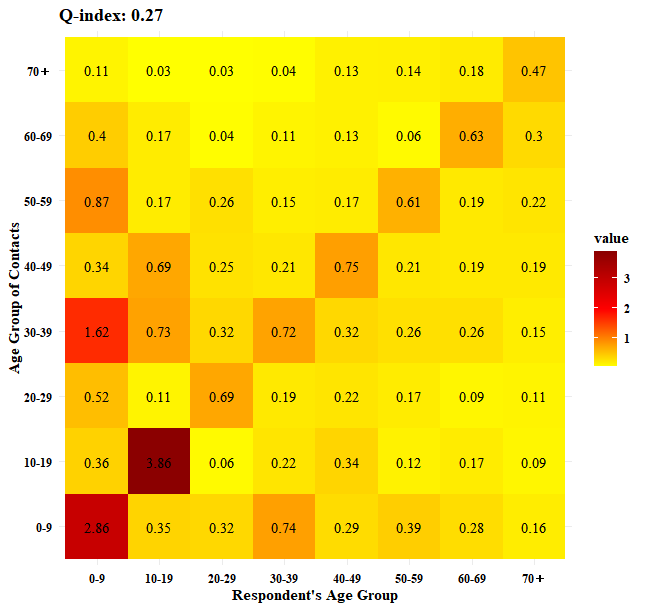


Figure S8. Non-physical contact matrix of reported contacts consisting of the average number of contacts per day per participant.


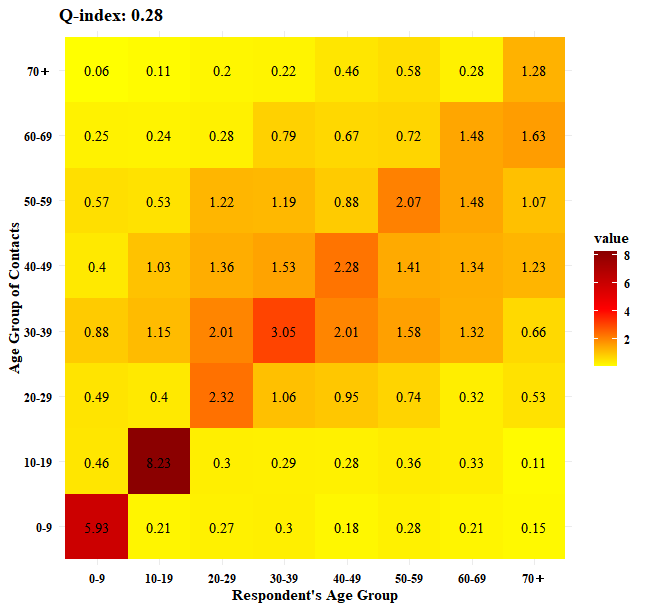


Figure S9. Contact matrix of reported contacts consisting of the average number of contacts per day per participant in weekday.


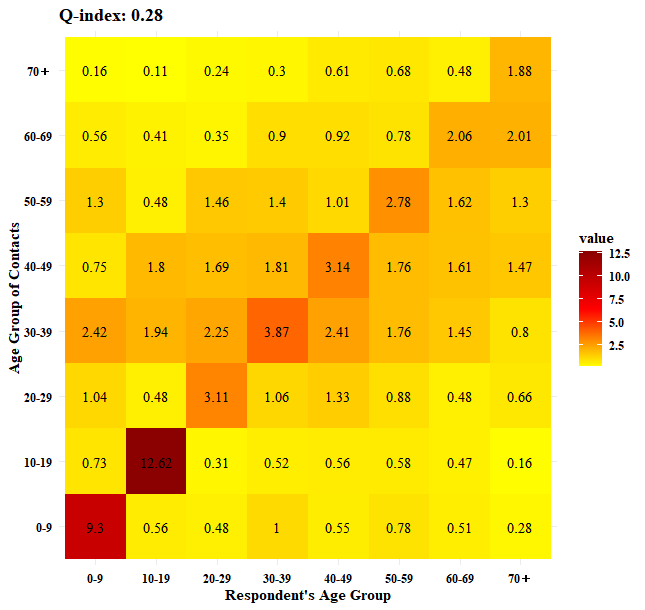


Figure S10. Contact matrix of reported contacts consisting of the average number of contacts per day per participant in weekend.


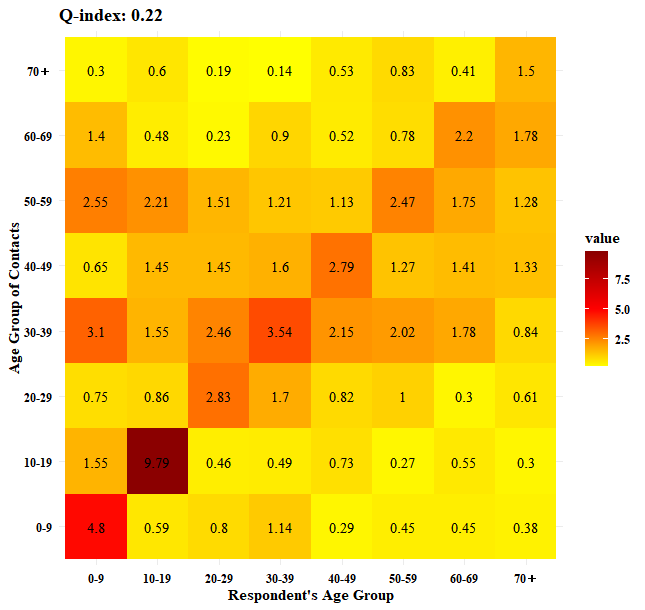

Supplement: Supplementary file 2 — Supplementary Material 2 [file 41043_2024_555_MOESM2_ESM.docx]
