# Supplementary material for: Social contacts patterns relevant to the transmission of infectious diseases in Suzhou, China following the COVID-19 epidemic
Source: J Health Popul Nutr. 2024 May 9;43:58. doi: 10.1186/s41043-024-00555-x (PMC11080078; doi:10.1186/s41043-024-00555-x)
Supplement: Supplementary file 3 — Supplementary Material 3 [file 41043_2024_555_MOESM3_ESM.docx]

**Supplementary Text. Sample paper questionnaire in English**

We showed only two contact entries in PART 2 SOCIAL CONTACT DIARY. There were 20 entries in the actual paper questionnaire we provided to the participants.

**Investigation on social contact patterns related to the spread of infectious diseases in Suzhou, China**

**PART 1: DEMOGRAPHIC DATA**

(Please tick the appropriate option☑)

1. Age: _______years
2. Sex: □Male □Female
3. Please select your working status: □Full-time □Part-time

Please select your working role:

□Public institution □Service □Enterprise □Worker □Student

□individual operation □[homemaker](javascript:%20void(0)) □Retired □Others

1. Highest level of education attained (completed):

□Primary school and below □Middle school □High school □college

□university □postgraduate □Ph.D. and above

1. Please select to provide your individual OR household monthly income (RMB):

□Individual income □Household income

Please provide the range of monthly income:

□0-5,999 □6,000-9,999 □10,000-14,999 □15,000-19,999 □20,000-24,999

□25,000-29,999 □30,000-39,999 □40,000-59,999 □60,000-79,999 □80,000-99,999

□>100,000

1. Place of birth: □Suzhou □Other parts of Jiangsu Province □Outside the province
2. District you are living in:

□High-tech Zone □Wuzhong district □Gusu district □Xiangcheng district

□Wujiang district □Industrial Park □Changshu city □Zhangjiagang city

□Kunshan city □Taicang city □Others

1. Nationality:

□Han □Zhuang □Hui □Manchus □Xinjiang Uygur □Miao □Others

1. Are you a Suzhou permanent resident: □Yes □No
2. How many people do you live with (sleep in the place more than 4 nights per week)?

Number of persons: _______persons

Type: □Household □Student residential hall □Shared apartment

**PART 2: SOCIAL CONTACT DIARY**

**INSTRUCTIONS**

1) Record in the contact diary every person you have contact with on your assigned day, regardless of whether the

contact was long or short, and whether you know the person or not.

2) A contact is defined as:

• EITHER skin-to-skin contact (physical contact, such as a handshake, hug, kiss)

• OR face-to-face conversation with three or more words exchanged, within 1-2 meters (non-physical contact).

Ignore conversations made over phones or on computers.

3) If you contact the same person several times during the assigned day, only record him/her once, and record the total time you spent with that person over the entire day. So, each person you meet during the day and have contact with should have one line in the diary.

11. The actual date you fill in the questionnaire:____(DD) / ____(MM)

12. For the purposes of this study, the day starts at 5 a.m. on the morning and ends at 5 a.m. the next morning.

Provide contact details by filling in the table:

| # | Contact demographics | Contact details | | Record time | How often do you have  contact with this person? |
| --- | --- | --- | --- | --- | --- |
| 1 | Age: □0-9 □10-19  □20-29□30-39  □40-49□50-59  □60-69□>=70 | Contact type  □physical contact  □non-physical contact | | ____：____ | □Daily or almost daily  □Once or twice a week  □Once or twice a month  □Less than once a month  □Never meet before |
|  |  | Total time：  □<5mins  □5-14mins  □15-59mins  □1-4hrs  □>4hrs | Where：  □House  □Work  □School  □Transport  □Others |  |  |
|  | Sex：□Male □Female |  |  |  |  |
| 2 | Age: □0-9 □10-19  □20-29□30-39  □40-49□50-59  □60-69□>=70 | Contact type  □physical contact  □non-physical contact | | ____：____ | □Daily or almost daily  □Once or twice a week  □Once or twice a month  □Less than once a month  □Never meet before |
|  |  | Total time：  □<5mins  □5-14mins  □15-59mins  □1-4hrs  □>4hrs | Where：  □House  □Work  □School  □Transport  □Others |  |  |
|  | Sex：□Male □Female |  |  |  |  |

Please make sure you have not left out any contacts.

13. Have you included all contacts?

□Yes

□No, approximately how many people you have left out: _______persons
